# Supplementary material for: Molecular Markers Associated with Agro-Physiological Traits under Terminal Drought Conditions in Bread Wheat
Source: Int J Mol Sci. 2020 Apr 30;21(9):3156. doi: 10.3390/ijms21093156 (PMC7247584; doi:10.3390/ijms21093156)
Supplement: Supplementary file 1 [file ijms-21-03156-s001.pdf]

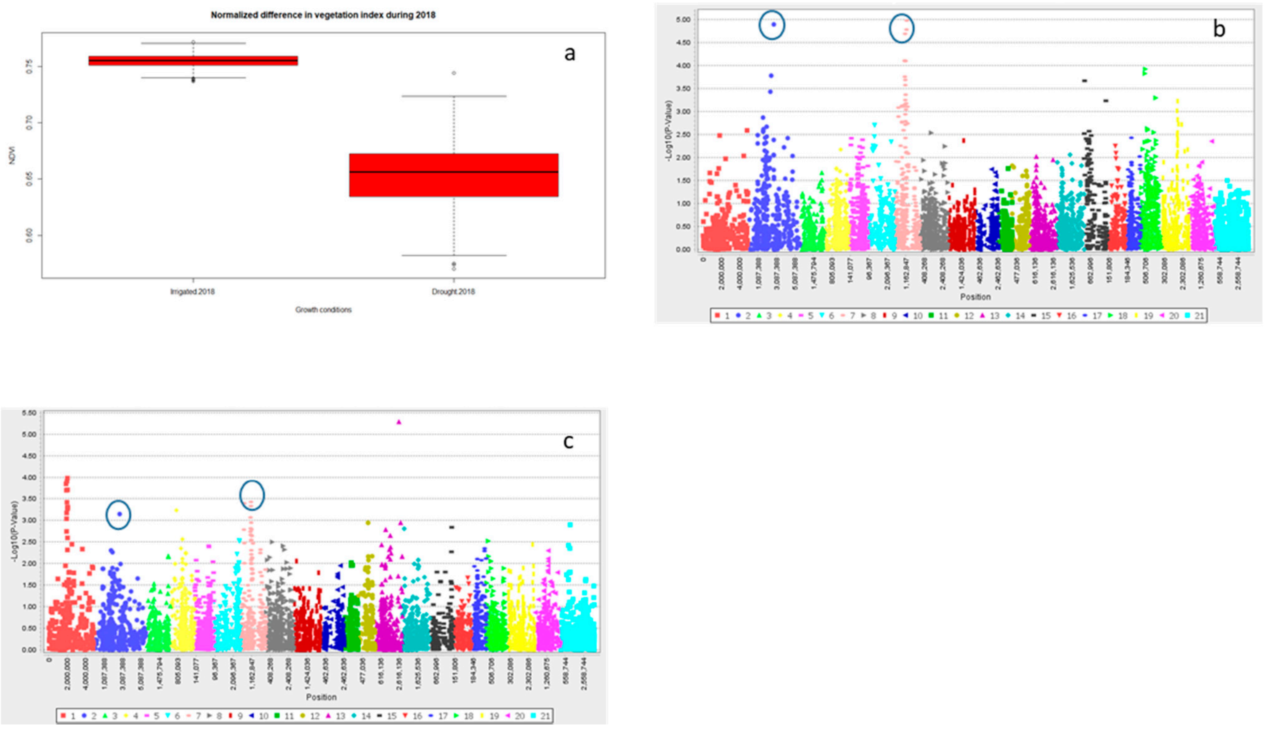

Figure S1. Boxplot showing variation in normalized difference in vegetative index (a). Marker trait association through GLM under irrigated (b) and drought (c) conditions

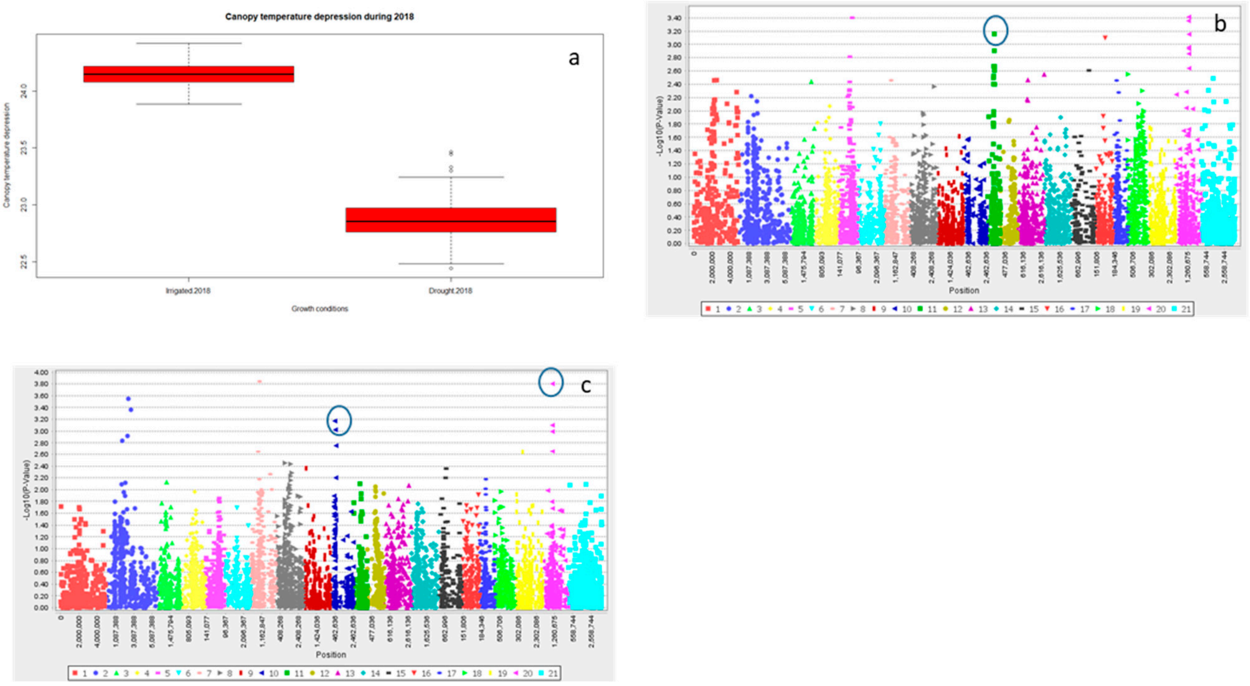

Figure S2. Boxplot showing variation in canopy temperature depression during 2018 (a). marker trait association through GLM under irrigated 2018 (b) and drought (c) conditions 2018

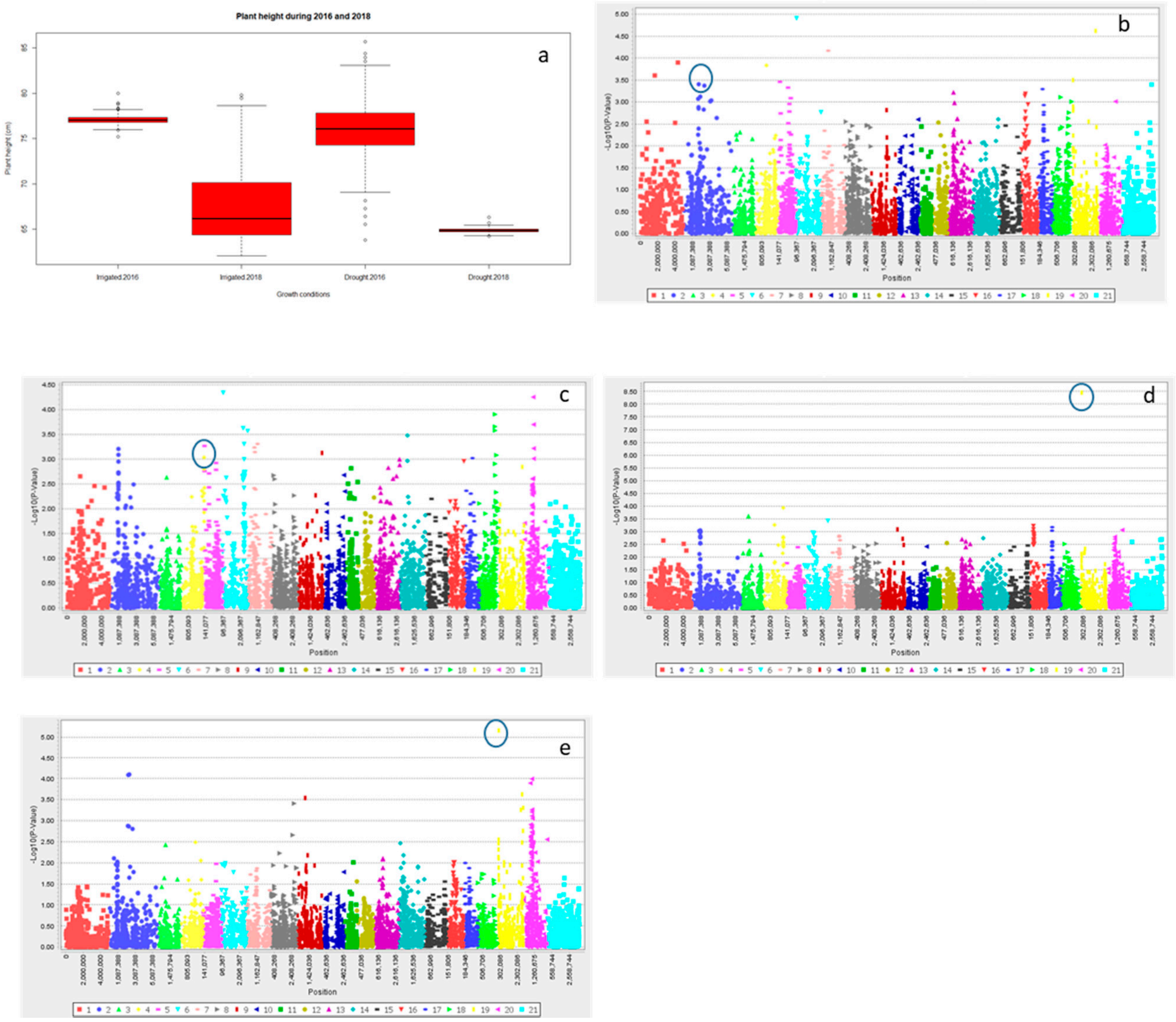

Figure S3. Boxplot showing variation for plant height under irrigated conditions 2016 and 2018 and drought condition for 2016 and 2018 (a). Marker trait association through GLM under irrigated (b) and drought (c) conditions 2016 and; irrigated (d) and drought conditions (e) 2018

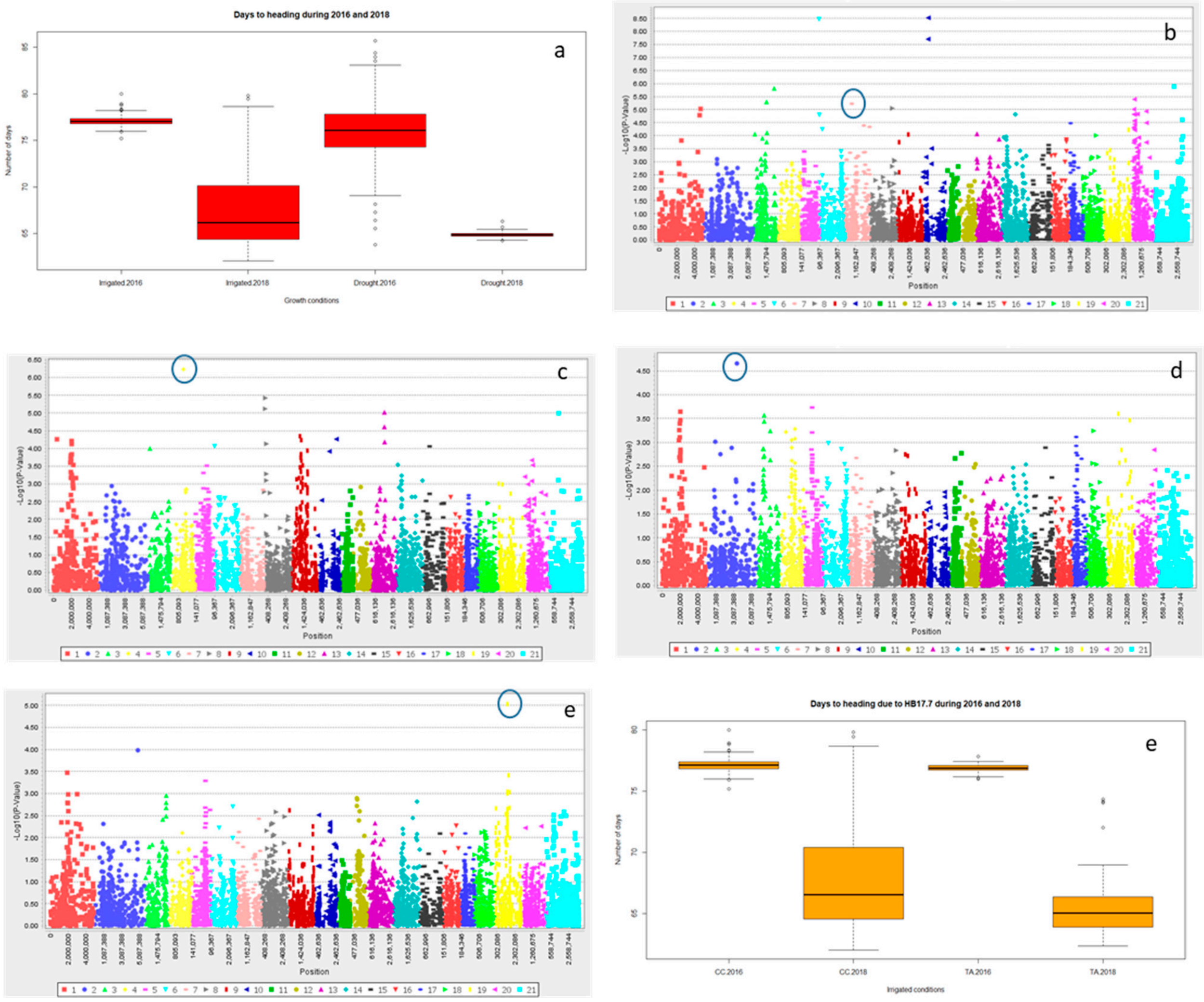

Figure S4. Boxplot showing variation for days to heading under irrigated conditions 2016 and 2018 and drought condition for 2016 and 2018 (a). Marker trait association through GLM under irrigated (b) and drought (c) conditions 2016 and; irrigated 2018 (d) and drought (e) conditions 2018. HB 7.17 showing differences in days to heading under irrigated condition for 2016 and 2018 (f).

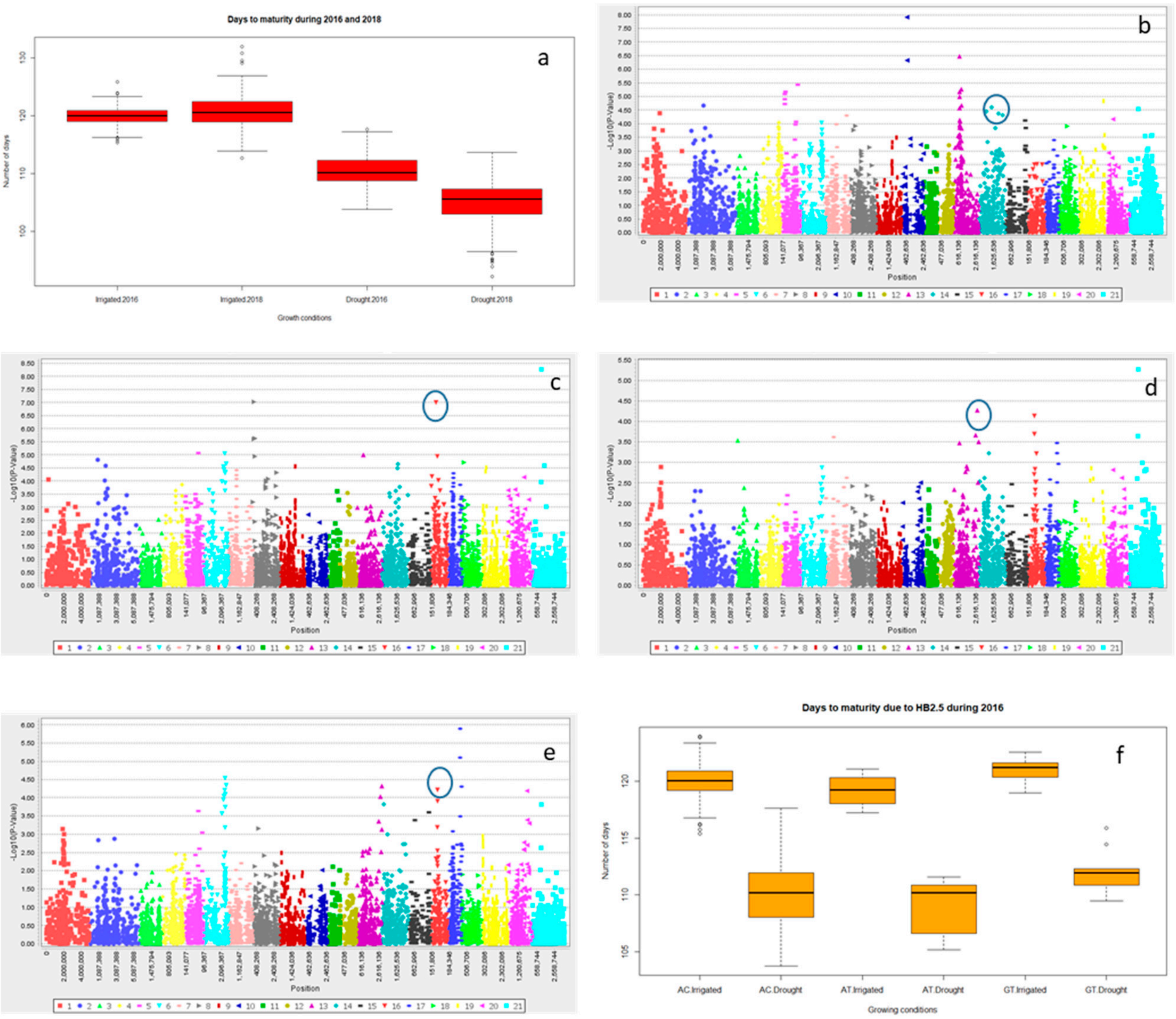

Figure S5. Boxplot showing variation for days to maturity under irrigated conditions 2016 and 2018 and drought condition for 2016 and 2018 (a). Marker trait association through GLM under irrigated (b) and drought conditions 2016 (c) and; irrigated (d) and drought (e) conditions 2018. HB 2.5 showing differences in days to maturity under irrigated condition for 2016 and 2018 (f).

International Journal of
Molecular Sciences

MDPI

Table- S1 Mix linear model describing the location of different traits on chromosomes

Year	Treatment	Trait	Marker	Chromosome	Log10	P-value	R^2 (%)
2018	Irrigated	NDVI	1216643	3A	4.32	4.83e-05	5.4
	Drought		1083852	3A	3.07	8.57e-04	3.7
2018	Irrigated	NDVI	4910039	1B	4.08	8.28e-05	5.0
	Drought		4910039	1B	3.09	8.16e-04	3.6
2018	Irrigated	CTD	1120870	4B	3.14	7.31e-04	3.7
	Drought		2257794	7B	3.46	3.47e-04	4.3
2018	Drought		1007667	4A	3.04	9.06e-04	3.5
2016	Irrigated	PH	1114828	1B	4.35	4.47e05	5.6
	Drought		1099561	2A	3.82	1.50e-04	5.2
2018	Irrigated		990180	7A	6.80	1.58e-07	10.3
	Drought		990180	7A	4.36	4.36e-05	6.2
2016	Irrigated	DTH	1084778	3A	3.88	1.31e-04	5.4
	Drought		1201200	2A	5.8	1.58e-06	8.8
2018	Irrigated		1056260	1B	3.92	1.19e-04	6.2
	Drought		1094037	7A	4.53	2.96e-05	5.6
2016	Irrigated	DTM	1106692	5B	4.6	7.56e-05	5.6
	Drought		4910893	6A	5.22	6.04e-06	6.9
2018	Irrigated		2261896	5A	4.12	7.56e-05	5.2
	Drought		4910893	6A	4.42	3.82e-05	5.8

NDVI = Normalized difference vegetation index, CTD = Canopy temperature depression, PH = Plant height, DTH = Days to heading and DTM = Days to maturity

Table S2: Grain yield in different wheat genotypes under drought conditions in response to favorable and unfavorable allele of haplotype block (HB)10.7 at chromosome 4A

GIDs-GBS	Pedigree	Pedigree	HB10.7	Y -D-2016	Y -D-2018	Ave-Y
7642492	CHEN/AE.SQ//WEAVER/3/VILLA JUAREZ F2009/4/WBLL1/KUKUNA//TACUPETO F2001/3/BAJ #1	SDSS12B00887T-0Y-0B-0B-37Y-0M	AC	2962.09	4606.86	3784.48
7643080	CHEN/AE.SQ//2*OPATA/5/SERI.1B//KAUZ/HEVO/3/AMAD*2/4/KIRITATI/6/FRET2*2/SHAMA//KACHU	SDSS12B00920T-0Y-0B-0B-68Y-0M	AC	2707.2	4814.29	3760.74
7643357	IG 41514/5/SERI.1B//KAUZ/HEVO/3/AMAD*2/4/KIRITATI/6/FRET2*2/SHAMA//KACHU	SDSS12B00927T-0Y-0B-0B-65Y-0M	AC	2809.96	4628.9	3719.43
7643355	IG 41514/5/SERI.1B//KAUZ/HEVO/3/AMAD*2/4/KIRITATI/6/FRET2*2/SHAMA//KACHU	SDSS12B00927T-0Y-0B-0B-63Y-0M	AC	2713.88	4704.57	3709.22
7645368	IG 107117/BORL14//COPIO	SDSS12B01122T-0Y-0B-0B-26Y-0M	AC	2729.89	4686.8	3708.35
7642611	IG 122793/VILLA JUAREZ F2009/4/WBLL1/KUKUNA//TACUPETO F2001/3/BAJ #1	SDSS12B00891T-0Y-0B-0B-22Y-0M	AC	2644.48	4693.82	3669.15
7645143	D67.2/PARANA 66.270//AE.SQUARROSA (301)/3/NELOKI/4/ATTILA*2/PBW65//MURGA	SDSS12B01094T-0Y-0B-0B-17Y-0M	AC	2787.27	4548.55	3667.91
7643448	IG 131673/5/SERI.1B//KAUZ/HEVO/3/AMAD*2/4/KIRITATI/6/FRET2*2/SHAMA//KACHU	SDSS12B00934T-0Y-0B-0B-101Y-0M	AC	2625.8	4653.02	3639.41
7642958	ALTAR 84/AE.SQUARROSA (1068)/4/WBLL1/KUKUNA//TACUPETO F2001/3/BAJ #1/5/SERI.1B//KAUZ/HEVO/3/AMAD*2/4/KIRITATI	SDSS12B00904T-0Y-0B-0B-28Y-0M	AC	2449.64	4788.9	3619.27
7642491	CHEN/AE.SQ//WEAVER/3/VILLA JUAREZ F2009/4/WBLL1/KUKUNA//TACUPETO F2001/3/BAJ #1	SDSS12B00887T-0Y-0B-0B-36Y-0M	AC	2421.62	4806.97	3614.29
7644625	68.111/RGB-U//WARD/3/FGO/4/RABI/5/AE.SQUARROSA (878)/6/KAUZ//ALTAR 84/AOS/3/<br>PASTOR/4/MILAN/CUPE//SW89.3064/5/KIRITATI/7/SW89.5277/BORL95//SKAUZ/3/PRL/2*PASTOR/4/HEILO	SDSS12B01031T-0Y-0B-0B-58Y-0M	AC	2465.66	4755.32	3610.49
7643346	IG 41514/5/SERI.1B//KAUZ/HEVO/3/AMAD*2/4/KIRITATI/6/FRET2*2/SHAMA//KACHU	SDSS12B00927T-0Y-0B-0B-54Y-0M	AC	2605.78	4612.26	3609.02
7642000	IG 42161/BAJ #1//SUP152	SDSS12B00872T-0Y-0B-0B-22Y-0B	AC	2709.87	4463.19	3586.53
7644199	68.111/RGB-U//WARD/3/AE.SQUARROSA (388)/7/SHA7/VEE#5/5/VEE#8//JUP/BJY/3/F3.71/TRM/4/2*WEAVER/6/SKAUZ/PARUS//PARUS/8/<br>CNDO/R143//ENTE/MEXI_2/3/AEGILOPS SQUARROSA (TAUS)/4/WEAVER/5/PICUS/6/TROST/7/TACUPETO F2001	SDSS12B00997T-0Y-0B-0B-20Y-0M	AC	2712.54	4456.3	3584.42
7643438	IG 131673/5/SERI.1B//KAUZ/HEVO/3/AMAD*2/4/KIRITATI/6/FRET2*2/SHAMA//KACHU	SDSS12B00934T-0Y-0B-0B-91Y-0M	AC	2475	4679.1	3577.05
7643587	INDIA-227/5/SERI.1B//KAUZ/HEVO/3/AMAD*2/4/KIRITATI/6/FRET2*2/SHAMA//KACHU	SDSS12B00942T-0Y-0B-0B-40Y-0M	AC	2559.07	4590.66	3574.87
7644875	IG 41217/4/PRL/2*PASTOR//PBW343*2/KUKUNA/3/ROLF07/5/NELOKI	SDSS12B01065T-0Y-0B-0B-29Y-0M	AC	2716.54	4429.84	3573.19
7643588	INDIA-227/5/SERI.1B//KAUZ/HEVO/3/AMAD*2/4/KIRITATI/6/FRET2*2/SHAMA//KACHU	SDSS12B00942T-0Y-0B-0B-41Y-0M	AC	2619.13	4508.32	3563.72
7644235	ACO89/AE.SQUARROSA (290)/7/SHA7/VEE#5/5/VEE#8//JUP/BJY/3/F3.71/TRM/4/2*WEAVER/6/SKAUZ/PARUS//PARUS/8/<br>CNDO/R143//ENTE/MEXI_2/3/AEGILOPS SQUARROSA (TAUS)/4/WEAVER/5/PICUS/6/TROST/7/TACUPETO F2001	SDSS12B01001T-0Y-0B-0B-32Y-0M	AC	2559.07	4561.41	3560.24
7645371	IG 107117/BORL14//COPIO	SDSS12B01122T-0Y-0B-0B-29Y-0M	AC	2414.95	4674.79	3544.87
7643587	INDIA-227/5/SERI.1B//KAUZ/HEVO/3/AMAD*2/4/KIRITATI/6/FRET2*2/SHAMA//KACHU	SDSS12B00942T-0Y-0B-0B-40Y-0M	AC	2559.07	4590.66	3574.87
7644875	IG 41217/4/PRL/2*PASTOR//PBW343*2/KUKUNA/3/ROLF07/5/NELOKI	SDSS12B01065T-0Y-0B-0B-29Y-0M	AC	2716.54	4429.84	3573.19
7643588	INDIA-227/5/SERI.1B//KAUZ/HEVO/3/AMAD*2/4/KIRITATI/6/FRET2*2/SHAMA//KACHU	SDSS12B00942T-0Y-0B-0B-41Y-0M	AC	2619.13	4508.32	3563.72
7644235	ACO89/AE.SQUARROSA (290)/7/SHA7/VEE#5/5/VEE#8//JUP/BJY/3/F3.71/TRM/4/2*WEAVER/6/SKAUZ/PARUS//PARUS/8/<br>CNDO/R143//ENTE/MEXI_2/3/AEGILOPS SQUARROSA (TAUS)/4/WEAVER/5/PICUS/6/TROST/7/TACUPETO F2001	SDSS12B01001T-0Y-0B-0B-32Y-0M	AC	2559.07	4561.41	3560.24
7645371	IG 107117/BORL14//COPIO	SDSS12B01122T-0Y-0B-0B-29Y-0M	AC	2414.95	4674.79	3544.87
7643458	IG 131673/5/SERI.1B//KAUZ/HEVO/3/AMAD*2/4/KIRITATI/6/FRET2*2/SHAMA//KACHU	SDSS12B00934T-0Y-0B-0B-111Y-0M	AC	2557.74	4531.9	3544.82
7642710	BCN//SORA/AE.SQUARROSA (323)/4/WBLL1/KUKUNA//TACUPETO F2001/3/BAJ #1/5/SERI.1B//KAUZ/HEVO/3/AMAD*2/4/KIRITATI	SDSS12B00909T-0Y-0B-0B-50Y-0M	AC	2428.29	4651.83	3540.06

7643081	CHEN/AE.SQ//2*OPATA/5/SERI.1B//KAUZ/HEVO/3/AMAD*2/4/KIRITATI/6/FRET2*2/SHAMA//KACHU	SDSS12B00920T-0Y-0B-0B-69Y-0M	AC	2668.5	4410.17	3539.33
**7644049**	**D67.2/PARANA 66.270//AE.SQUARROSA (465)/3/WHEAR/KRONSTAD F2004/7/SHA7/VEE#5/5/VEE#8//JUP/BJY/3/F3.71/TRM/4/2*WEAVER/6/SKAUZ/PARUS//PARUS**	**SDSS12B00979T-0Y-0B-0B-18Y-0M**	**AC**	**2575.09**	**4503.35**	**3539.22**
7641976	CHEN/AE.SQ//2*OPATA/3/BAJ #1/4/SUP152	SDSS12B00870T-0Y-0B-0B-172Y-0M	AC	2428.29	4647.8	3538.05
7644127	MEX94.13.1.47//WHEAR/KRONSTAD F2004/7/SHA7/VEE#5/5/VEE#8//JUP/BJY/3/F3.71/TRM/4/2*WEAVER/6/SKAUZ/PARUS//PARUS	SDSS12B00989T-0Y-0B-0B-15Y-0M	AC	2394.93	4678.35	3536.64
7642658	BCN//CETA/AE.SEARSII (34D)/4/WBLL1/KUKUNA//TACUPETO F2001/3/BAJ #1/5/SERI.1B//KAUZ/HEVO/3/AMAD*2/4/KIRITATI	SDSS12B00907T-0Y-0B-0B-50Y-0M	AC	2290.84	4775.84	3533.34
7644158	OAX93.5.1.1//WHEAR/KRONSTAD F2004/7/SHA7/VEE#5/5/VEE#8//JUP/BJY/3/F3.71/TRM/4/2*WEAVER/6/SKAUZ/PARUS//PARUS	SDSS12B00990T-0Y-0B-0B-47Y-0M	AC	2420.29	4644.52	3532.4
7645338	IG 41624/BORL14//COPIO	SDSS12B01120T-0Y-0B-0B-13Y-0M	AC	2609.78	4447.27	3528.53
**7644071**	**SHAG_22/AE.SQUARROSA (721)//WHEAR/KRONSTAD F2004/7/SHA7/VEE#5/5/VEE#8//JUP/BJY/3/F3.71/TRM/4/2*WEAVER/6/SKAUZ/PARUS//PARUS**	**SDSS12B00981T-0Y-0B-0B-22Y-0M**	**AC**	**2389.59**	**4665.02**	**3527.31**
7641715	CROC_1/AE.SQUARROSA (298)//KACHU/3/BAJ #1	SDSS12B00850T-0Y-0B-0B-99Y-0M	CT	2297.51	3998.24	3147.88
7641281	GAN/AE.SQUARROSA (897)//KACHU/3/BAJ #1	SDSS12B00826T-0Y-0B-0B-34Y-0M	CT	2077.32	4217.08	3147.2
7640870	IG 41475/KACHU//BAJ #1	SDSS12B00852T-0Y-0B-0B-48Y-0M	CT	2086.66	4173.9	3130.28
**7644054**	**D67.2/PARANA 66.270//AE.SQUARROSA (465)/3/WHEAR/KRONSTAD F2004/7/SHA7/VEE#5/5/VEE#8//JUP/BJY/3/F3.71/TRM/4/2*WEAVER/6/SKAUZ/PARUS//PARUS**	**SDSS12B00979T-0Y-0B-0B-23Y-0M**	**CT**	**2378.92**	**3875.14**	**3127.03**
7641448	CETA/AE.SQUARROSA (1025)//KACHU/3/BAJ #1	SDSS12B00835T-0Y-0B-0B-73Y-0M	CT	2042.62	4207.86	3125.24
7641562	D67.2/PARANA 66.270//AE.SQUARROSA (185)/3/KACHU/4/BAJ #1	SDSS12B00840T-0Y-0B-0B-31Y-0M	CT	1783.73	4446.09	3114.91
7640766	CETA/AE.SQUARROSA (665)//KACHU/3/BAJ #1	SDSS12B00822T-0Y-0B-0B-25Y-0M	CT	2262.81	3937.5	3100.16
7640992	BOTNO/AE.SQUARROSA (666)//KACHU/3/BAJ #1	SDSS12B00823T-0Y-0B-0B-34Y-0M	CT	2328.2	3850.85	3089.53
7642379	SORA/AE.SQUARROSA (442)//SUP152/3/VILLA JUAREZ F2009	SDSS12B00879T-0Y-0B-0B-29Y-0M	CT	2126.7	4045.59	3086.14
7641303	CROC_1/AE.SQUARROSA (516)//KACHU/3/BAJ #1	SDSS12B00832T-0Y-0B-0B-48Y-0M	CT	2200.09	3965.83	3082.96
7644024	T.DICOCCON CI3686/AE.SQUARROSA (458)//WHEAR/KRONSTAD F2004/7/SHA7/VEE#5/5/VEE#8//JUP/BJY/3/F3.71/TRM/4/2*WEAVER/6/SKAUZ/PARUS//PARUS	SDSS12B00978T-0Y-0B-0B-33Y-0M	CT	2129.36	4015.71	3072.54
7641307	CROC_1/AE.SQUARROSA (516)//KACHU/3/BAJ #1	SDSS12B00832T-0Y-0B-0B-52Y-0M	CT	2066.64	4076.86	3071.75
**7644069**	**SHAG_22/AE.SQUARROSA (721)//WHEAR/KRONSTAD F2004/7/SHA7/VEE#5/5/VEE#8//JUP/BJY/3/F3.71/TRM/4/2*WEAVER/6/SKAUZ/PARUS//PARUS**	**SDSS12B00981T-0Y-0B-0B-20Y-0M**	**CT**	**2169.4**	**3972.54**	**3070.97**
7644100	H-1534//WHEAR/KRONSTAD F2004/7/SHA7/VEE#5/5/VEE#8//JUP/BJY/3/F3.71/TRM/4/2*WEAVER/6/SKAUZ/PARUS//PARUS	SDSS12B00987T-0Y-0B-0B-39Y-0M	CT	1925.19	4216	3070.59
7640964	SCOOP_1/AE.SQUARROSA (634)//KACHU/3/BAJ #1	SDSS12B00821T-0Y-0B-0B-41Y-0M	CT	2057.3	3969.68	3013.49
7640871	IG 41475/KACHU//BAJ #1	SDSS12B00852T-0Y-0B-0B-49Y-0M	CT	2302.85	3690.56	2996.7
7641461	ARLIN_1/AE.SQUARROSA (1017)//KACHU/3/BAJ #1	SDSS12B00837T-0Y-0B-0B-49Y-0M	CT	2157.39	3823.16	2990.28
7641377	CROC_1/AE.SQUARROSA (517)//KACHU/3/BAJ #1	SDSS12B00833T-0Y-0B-0B-86Y-0M	CT	1811.75	4160.43	2986.09
7641835	H-1442/KACHU//BAJ #1	SDSS12B00859T-0Y-0B-0B-40Y-0B	CT	2073.32	3891.54	2982.43

7641585	CPI8/GEDIZ/3/GOO//ALB/CRA/4/AE.SQUARROSA (227)/5/KACHU/6/BAJ #1	SDSS12B00841T-0Y-0B-0B-28Y-0M	CT	2021.27	3920.73	2971
7643943	DOY1/AE.SQUARROSA (532)//WHEAR/KRONSTAD F2004/7/SHA7/VEE#5/5/<br>VEE#8//JUP/BJY/3/F3.71/TRM/4/2*WEAVER/6/SKAUZ/PARUS//PARUS	SDSS12B00971T-0Y-0B-0B-25Y-0M	CT	1947.87	3946.22	2947.05
7642168	DOY1/AE.SQUARROSA (264)//BAJ #1/3/SUP152	SDSS12B00869T-0Y-0B-0B-47Y-0M	CT	1887.82	3934.74	2911.28
7643948	DOY1/AE.SQUARROSA (532)//WHEAR/KRONSTAD F2004/7/SHA7/VEE#5/5/<br>VEE#8//JUP/BJY/3/F3.71/TRM/4/2*WEAVER/6/SKAUZ/PARUS//PARUS	SDSS12B00971T-0Y-0B-0B-30Y-0M	CT	2096	3718.81	2907.41
7640991	BOTNO/AE.SQUARROSA (666)//KACHU/3/BAJ #1	SDSS12B00823T-0Y-0B-0B-33Y-0M	CT	1679.64	4073.77	2876.7
7645476	DOY1/AE.SQUARROSA (318)/3/KACHU #1/KIRITATI//KACHU/4/PBW343*2/KUKUNA*2//FRTL/PIFED	SDSS12B01135T-0Y-0B-0B-19Y-0M	CT	1847.78	3884.7	2866.24
7641495	CETA/AE.SQUARROSA (1055)//KACHU/3/BAJ #1	SDSS12B00838T-0Y-0B-0B-42Y-0M	CT	1954.54	3712.84	2833.69
7643965	ALTAR 84/AE.SQUARROSA (502)//WHEAR/KRONSTAD F2004/7/SHA7/VEE#5/5/<br>VEE#8//JUP/BJY/3/F3.71/TRM/4/2*WEAVER/6/SKAUZ/PARUS//PARUS	SDSS12B00972T-0Y-0B-0B-15Y-0M	CT	2089.33	3557.84	2823.59
7641490	ARLIN_1/AE.SQUARROSA (1017)//KACHU/3/BAJ #1	SDSS12B00837T-0Y-0B-0B-78Y-0M	CT	2182.74	3394.69	2788.72
7644025	T.DICOCCON CI3686/AE.SQUARROSA (458)//WHEAR/KRONSTAD F2004/7/SHA7/VEE#5/5/<br>VEE#8//JUP/BJY/3/F3.71/TRM/4/2*WEAVER/6/SKAUZ/PARUS//PARUS	SDSS12B00978T-0Y-0B-0B-34Y-0M	CT	2116.02	3401.93	2758.97
7641450	CETA/AE.SQUARROSA (1025)//KACHU/3/BAJ #1	SDSS12B00835T-0Y-0B-0B-75Y-0M	CT	1878.48	3634.19	2756.33
7640782	CROC_1/AE.SQUARROSA (466)//KACHU/3/BAJ #1	SDSS12B00829T-0Y-0B-0B-50Y-0M	CT	2086.66	3404	2745.33
7640936	IG 42158/KACHU//BAJ #1	SDSS12B00855T-0Y-0B-0B-41Y-0M	CT	1818.43	3579.47	2698.95
7643973	ALTAR 84/AE.SQUARROSA (502)//WHEAR/KRONSTAD F2004/7/SHA7/VEE#5/5/<br>VEE#8//JUP/BJY/3/F3.71/TRM/4/2*WEAVER/6/SKAUZ/PARUS//PARUS	SDSS12B00972T-0Y-0B-0B-23Y-0M	CT	2007.92	3366.75	2687.34
7641264	TK SN1081/AE.SQUARROSA (690)//KACHU/3/BAJ #1	SDSS12B00824T-0Y-0B-0B-51Y-0M	CT	1795.74	3372.81	2584.27
7641502	CETA/AE.SQUARROSA (1055)//KACHU/3/BAJ #1	SDSS12B00838T-0Y-0B-0B-49Y-0M	CT	1967.89	2870.83	2419.36

Y-D-2016 = grain yield under drought during 2016, Y-D-2018 = grain yield under drought during 2018, Ave-Y= Average of grain yield under drought in 2016 and 2018 and yellow color highlights the genotypes with same parentage and pedigree but with different allele and yield

Table S3: Genotypes with minimum and maximum reduction in the yield and yield related traits due to favorable and unfavorable allele at chromosome 4A (HB 10.7)

Genotypes with minimum average reduction in Y under drought and with favorable haplotypes							
GID	Parentage	Pedigree	Y (%)	SL (%)	NGS (%)	TKW (%)	HB10.7
7645269	GARZA/BOY//AE.SQUARROSA (179)/3/ATTILA*2/PBW65//MURGA/4/BORL14	SDSS12B01105T-0Y-0B-0B-31Y-0M	29.98	5.32	12.5	8.64	AC
7643448	IG 131673/5/SERI.1B//KAUZ/HEVO/3/AMAD*2/4/KIRITATI/6/FRET2*2/SHAMA//KACHU	SDSS12B00934T-0Y-0B-0B-101Y-0M	29.76	0	11.86	0	AC
7645262	GARZA/BOY//AE.SQUARROSA (179)/3/ATTILA*2/PBW65//MURGA/4/BORL14	SDSS12B01105T-0Y-0B-0B-24Y-0M	29.55	0.08	6.91	13.18	AC
7645186	INDIA-46/NELOKI/3/ATTILA*2/PBW65//MURGA	SDSS12B01098T-0Y-0B-0B-22Y-0M	28.935	0	8.53	11.32	AC
7643357	IG 41514/5/SERI.1B//KAUZ/HEVO/3/AMAD*2/4/KIRITATI/6/FRET2*2/SHAMA//KACHU	SDSS12B00927T-0Y-0B-0B-65Y-0M	28.89	1	10.12	5.13	AC
7645251	CETA/AE.SQUARROSA (533)/3/ATTILA*2/PBW65//MURGA/4/BORL14	SDSS12B01104T-0Y-0B-0B-10Y-0M	28.77	0	14.12	5.22	AC
7645338	IG 41624/BORL14//COPIO	SDSS12B01120T-0Y-0B-0B-13Y-0M	28.65	6.14	1.09	14.13	AC
7645368	IG 107117/BORL14//COPIO	SDSS12B01122T-0Y-0B-0B-26Y-0M	28.63	6.8	4.23	15.32	AC
7642492	CHEN/AE.SQ//WEAVER/3/VILLA JUAREZ F2009/4/WBLL1/KUKUNA//TACUPETO F2001/3/BAJ #1	SDSS12B00887T-0Y-0B-0B-37Y-0M	28.02	8.3	3.92	7.82	AC
7645268	GARZA/BOY//AE.SQUARROSA (179)/3/ATTILA*2/PBW65//MURGA/4/BORL14	SDSS12B01105T-0Y-0B-0B-30Y-0M	27.61	0	9.55	0	AC
7645258	GARZA/BOY//AE.SQUARROSA (179)/3/ATTILA*2/PBW65//MURGA/4/BORL14	SDSS12B01105T-0Y-0B-0B-20Y-0M	26.7	7.5	5.69	10.22	AC
7645143	D67.2/PARANA 66.270//AE.SQUARROSA (301)/3/NELOKI/4/ATTILA*2/PBW65//MURGA	SDSS12B01094T-0Y-0B-0B-17Y-0M	26.64	0.9	17.9	0	AC
7643713	MAYOOR//TK SN1081/AE.SQUARROSA (222)/3/FRET2*2/SHAMA//KACHU/4/HUW234+LR34/PRINIA*2//KIRITATI	SDSS12B00950T-0Y-0B-0B-20Y-0M	26.46	0	7.57	8.03	AC
7645073	TXL92.7.5.1/4/PRL/2*PASTOR//PBW343*2/KUKUNA/3/ROLF07/5/NELOKI	SDSS12B01090T-0Y-0B-0B-17Y-0M	25.75	0	8.66	9.14	AC
Genotypes with maximum average reduction in Y under drought and with un-favorable haplotypes							
7641264	TK SN1081/AE.SQUARROSA (690)//KACHU/3/BAJ #1	SDSS12B00824T-0Y-0B-0B-51Y-0M	46.44	7.44	8.1	11.95	CT
7640766	CETA/AE.SQUARROSA (665)//KACHU/3/BAJ #1	SDSS12B00822T-0Y-0B-0B-25Y-0M	43.66	10.98	9.24	17.08	CT
7640991	BOTNO/AE.SQUARROSA (666)//KACHU/3/BAJ #1	SDSS12B00823T-0Y-0B-0B-33Y-0M	43.38	5.71	8.67	12.64	CT
7641377	CROC_1/AE.SQUARROSA (517)//KACHU/3/BAJ #1	SDSS12B00833T-0Y-0B-0B-86Y-0M	42.98	0	15.37	2.71	CT
7641281	GAN/AE.SQUARROSA (897)//KACHU/3/BAJ #1	SDSS12B00826T-0Y-0B-0B-34Y-0M	42.77	9.9	9.76	15.96	CT
7641450	CETA/AE.SQUARROSA (1025)//KACHU/3/BAJ #1	SDSS12B00835T-0Y-0B-0B-75Y-0M	42.49	8.76	5.23	15.91	CT
7640823	KE90-282/MILAN//KACHU/3/BAJ #1	SDSS12B00849T-0Y-0B-0B-66Y-0M	42.32	0	7.93	5.06	CT

7641307	CROC_1/AE.SQUARROSA (516)//KACHU/3/BAJ #1	SDSS12B00832T-0Y-0B-0B-52Y-0M	42.11	9.97	4.5	16.64	CT
7641537	LOCAL RED/AE.SQUARROSA (189)//KACHU/3/BAJ #1	SDSS12B00839T-0Y-0B-0B-44Y-0M	42.02	5.42	4.02	15.74	CT
7640828	KE90-282/MILAN//KACHU/3/BAJ #1	SDSS12B00849T-0Y-0B-0B-71Y-0M	41.75	1.11	13.8	8.95	CT
7641303	CROC_1/AE.SQUARROSA (516)//KACHU/3/BAJ #1	SDSS12B00832T-0Y-0B-0B-48Y-0M	41.57	9.68	17.27	17.5	CT
7641490	ARLIN_1/AE.SQUARROSA (1017)//KACHU/3/BAJ #1	SDSS12B00837T-0Y-0B-0B-78Y-0M	41.28	0	14.41	0	CT

Y% = % reduction in grain yield, SL%= % reduction in spike length, NGS%= % reduction in number of grains spike^{-1}, TKW % = % reduction in thousand kernel weight, HB10.7 = haplotype block 10.7 at chromosome 4A

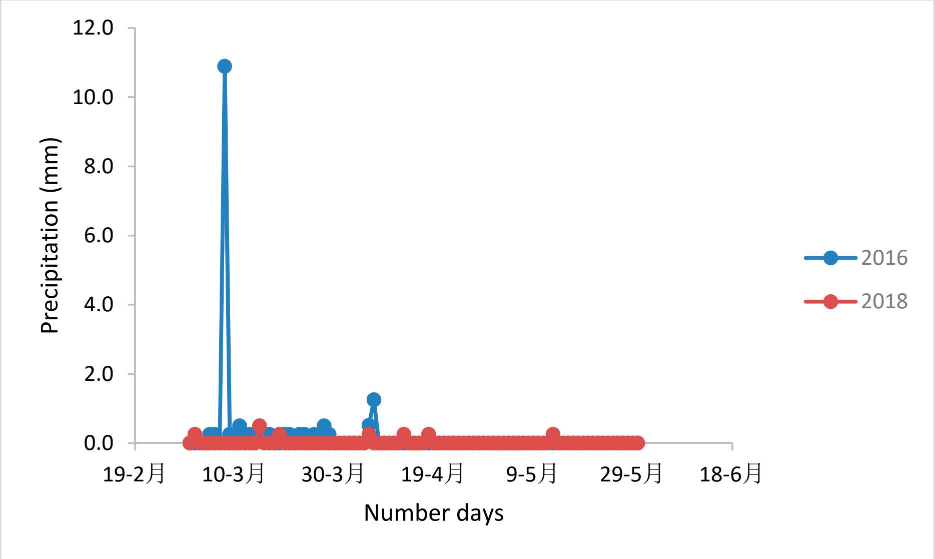

Figure S6: Precipitation after drought stress imposed during 2016 and 2018
